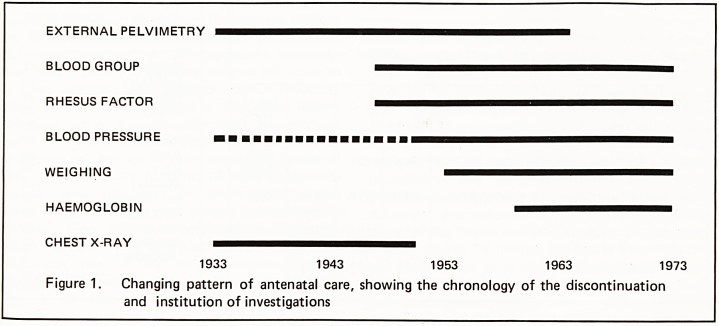# Forty Years of Obstetrics in General Practice

**Published:** 1977

**Authors:** Christopher Morgans


### Forty Years of Obstetrics in General Practice

**Christopher Morgans**

One man's experience of a lifetime of practice can be of little value to the sophisticated experts of today in obstetrics but may be of interest to those who have lived through a similar period of drastic changes. Prompted by a desire to produce something useful in retirement, I thought that a survey of my own experience of obstetrics in general practice over a period of forty years in Bristol might be of some interest and value. On reaching retirement at the age of 66 I had a mass of old pre-National Health Service maternity records, all on a private basis, together with records extracted from some NHS cases from present day files. There were however hundreds of 'lost' notes of patients who had moved away from Bristol, possibly half the total number over the years. This paper therefore is incomplete, limited, not representative, inaccurate, with many 'holes' in the records; it cannot be pressed too hard statistically; but it does perhaps provide a useful account of one man's changing pattern of care over forty years.

The series comprises 916 cases or 928 babies (there being 12 sets of twins), covering the years from 1933 to 1973, a period which saw a remarkable fall in both maternal and infant mortality rates, the former from about 400 to about 15 per 100,000; the latter from about 60 to about 20 per 1,000. The battle to reduce these figures still further continues using increasingly sophisticated mechanical devices; but at the same time many thousands of young lives are destroyed in abortions.

I emerged from St. Thomas's Hospital with certain ideals and principles which served as a basic approach to maternity work. These were as follows:

1. That when I put up my plate, the care of mothers and babies would be the backbone of the practice I hoped to build up and would probably account for three-quarters of the work.
2. That ante-natal care should start as early as possible in pregnancy, a careful history should be taken and full examination made then, possibly anticipating a threat of miscarriage. Thereafter she should be seen monthly till the eight month, then weekly or oftener till delivery. Urine would be tested for albumen and sugar on each occassion, but the blood pressure need only be repeated when albuminuria or oedema suggested toxaemia.
3. That the pelvis should be measured carefully externally, and that the fit of the baby's head into the pelvic brim should be assessed by careful palpation in the last few weeks; and that any disproportion suspected should be treated by inducing labour, at first by medical induction, and if this failed, by surgically rupturing the membranes to ensure that labour started while 'the fit' was reasonable.
4. That I as the doctor in charge should be present at every delivery if possible, and that the more cases I personally delivered myself, including forceps delivery if needed, the better chance I had of becoming a good midwife.
5. That the district midwives were the 'salt of the earth' and had home confinements highly organised, for in those days many mothers preferred home confinement; but that I must be alert and ready to call in consultant advice and transfer to hospital in good time if complications threatened.
6. That if no help was readily available it was up to me to get on with the job and do the best I could with what knowledge and tools I had, including the simple administration of anaesthetics in small quantities for relief of pain, or as a general anaesthetic for any manipulation or forceps delivery.
7. That forceps should be boiled up and ready if after two hours in the second stage there was no progress being made.
8. That the mother should be prepared physically and psychologically, including dealing with ignorance and fears. She should also be assured that her wishes and welfare came first and that we were, literally, on duty at all times. She was told that there was hard and painful work ahead, but that it was usually well within her powers and it could be made easier by her approach; prolonged and exhausting labour could be helped by tablets, injection or anaesthesics. She was instructed in the best use of gas and air machines and reminded that we as a team were concerned for her safety, her comfort and the safe delivery of her baby, always hoping for a natural labour as the best way of having a baby.
9. That pregnancy was the time for both doctor and midwife to build up a relationship of confidence and trust with the patient.

10. That breast-fed was truly best-fed for many reasons of bonding and cheapness and healthy babies, but that if for any reason it was unacceptable, or a failure or inadvisable, artificial feeding could be a good second.
11. That the best dilator of the cervix was the bag of forewaters cut off by the down-coming head, and that the longer this was intact the better the delivery.
12. That successful delivery involved an untorn perineum keeping stitches to a minimum for the sake of the mother's comfort, and anything more than half an inch tear of the fourchette was regarded as a failure.
13. That the left lateral position was greatly preferable to the lithotomy position, both from the point of view of the comfort of the mother, and for the successful control of the delivery of the head, the safety of the anaesthetic, and indeed for a successful forceps delivery.
14. That husbands can be very useful at home deliveries, supporting and encouraging and comforting the wife, tea-making, leg-holding and telephone-answering, and that they and the mother should be kept up to date with progress reports and explanations by both doctor and midwife.

Armed with this simple, homely, almost naive, and very old-fashioned set of principles, I will try and indicate how I fared and what changes have occurred over the years. These may be considered under the headings: The Place, The Preparation, The Position and The Pain.

### THE CHANGING EXPERIENCE

### PLACE OF CONFINEMENT

In the early years in Bristol a mother had a choice of a home confinement, entry into a private maternity home with her own doctor attending her, or admission to a maternity hospital where she was under a specialist. My cases were: home 205, private maternity homes 629, hospital 76. Many consultant obstetricians declared war on home confinements over the years, and the fall in home confinements has therefore been dramatic, i.e. from about 29% of all cases in 1964 to about 8% in 1972.

I was loyal to consultant advice to persuade mothers at accepted risk to book into hospital, but reluctant to accept that all primiparae were at special risk, and grateful that in many cases the consultant trusted me to give good home care. But of course like others I came to toe the line where risk was concerned and so the number of cases in GP maternity hospitals increased greatly in later years. In my series there were two maternal deaths, a primipara aged 22 in 1943 who died with eclampsia, and one aged 33 in 1948 who died the day after delivery of twins from 'haemorrhage and cardiac failure'. Both had been transferred to hospital, the former after delivery and the latter before delivery, but both might have stood a better chance of survival if they had been planned admissions as they certainly would be today.

The main loss to the woman under today's arrangements seems to me to be not only a loss of confidence and security, but of continuity of personal relationship. She is passed from one to another, and any ante-natal relationship will not proceed to her confinement. In 1975 Professor O'Driscoll (Royal College of Obstetricians & Gynaecologists, 1975) claimed that in Dublin National Maternity Hospital, where there are 7,500 births per annum in one Unit 'every woman in labour has her own personal nurse'. On being asked 'does that mean that they stay on for an indefinite time and that they do not change shifts?' He replied 'they stay on until they go off duty and then somebody else takes over'! I am aware of cases in which this has occurred only a few minutes before the birth takes place. It is difficult to see how concern for the mother and her wishes comes first in the modern set up. Yet David Attenborough on television recently (17.12.76) told us that the keeper of Delilah the gorilla was with her all through her pregnancy and so was accepted at the time of confinement, and if left alone at confinement she was very nervous! I feel that other 'Delilah's' are entitled to the same advantage, and that this at least was satisfied in the home confinements of the 'old days'.

### THE PREPARATION

Ante-natal care shows a changing pattern over the 40 years. This is shown in figure 1. The explanation of this is as follows:

1. PELVIMETRY

   The external measurements of the pelvis were regarded as an essential if rough guide to the adequacy of the pelvis, and the majority were measured up to 1960, then doubts were cast on its usefulness, and in 1963 we were advised to 'throw away our pelvimeters'.

Figure 1. Changing pattern of antenatal care, showing the chronology of the discontinuation and institution of investigations

2. BLOOD PRESSURE
There was no early emphasis on the need to watch this, apart from the initial examination unless toxaemia was suspected, until in 1952 we became alerted, and a gradual increase occurred until all cases were checked 10 or more times.

3. BLOOD GROUPING
I did not start this until 1947, but by 1955 three-quarters of the cases were grouped and by 1961 all cases.

4. RHESUS FACTOR
This was begun about the same time and by 1960 all cases were recorded.

5. HAEMOGLOBIN PERCENTAGE
Until 1959 careful assessment of conjunctival colour was the practice! Out of 907 over 38 years, 241 were estimated in the last 13 years, and in recent years both at the start and at the end of pregnancy. Despite the desired 100%, it seems from my records that the oral administration of iron would improve readings from 80% to 90% but not often any further.

6. WEIGHING
Weighing the mother each time did not start until 1953 and results showed that out of 416 cases regularly weighed 142 gained 2 stones (12.7 kg) or more, 15 of them reached 41 lbs (18.6 kg) increase!

7. THE QUICKENING DATE
I regarded this as a useful check on the expecting date and results showed that of 713 cases 479 quickened in the 18th or 19th weeks of pregnancy and that this linked usefully with the week of delivery.

8. CHEST X-RAYING
Up to 1950 it was a sin to omit this as a routine part of examination for tuberculosis; from then on it was sinful to expose to x-rays unless essential, for obvious reasons!

9. ANTE-NATAL PREPARATION
In 1937 I began to give special ante-natal advice, i.e. a typewritten sheet with homely advice covering all possible questions arising in pregnancy: and in 1950 I began to send cases for relaxation classes either to a private physiotherapist or to a hospital or clinic. In the series about 600 received my advice only, and about 300 went to classes of which 95 were privately given to small groups. An attempt has been made to plot relaxation against the length of the stages of labour. This shows a possible advantage in the number of those delivered in the first hour of the second stage (Table 1).

**POSITION**

As mentioned I was trained to deliver in the left lateral position, and this of course has been one of the biggest changes over the years, mainly due to the teaching in Bristol for students and nurses. In my experience many women prefer to 'bring down the head' working on their backs, but are relieved when turned into the left lateral position for delivery. Pain in the sacral area towards full dilatation is often very

Table 1 **RELAXATION AND LABOUR**
**LENGTH OF 2nd STAGE OF LABOUR**

| TYPE OF PREPARATION (HOURS) | < 1 | 1–2 | > 2 | No. of Cases |
|---|---|---|---|---|
| A. NO SPECIAL PREPARATION | 54% | 22% | 24% | 267 (100%) |
| B. A.N. ADVICE SHEET + DISCUSSION | 59% | 21% | 20% | 350 |
| C. RELAXATION TAUGHT | 63% | 15% | 22% | 201 |
| D. PRIVATE CLASSES | 65% | 11% | 24% | 94 |

Table 2 **TOTALS OF BIRTHS PER MONTH**

| MONTH OF | TOTAL |
|---|---|
| JANUARY | 74 |
| FEBRUARY | 76 |
| MARCH | 80 |
| APRIL | 80 |
| MAY | 82 |
| JUNE | 84 |
| JULY | 79 |
| AUGUST | 106 |
| SEPTEMBER | 67 |
| OCTOBER | 64 |
| NOVEMBER | 63 |
| DECEMBER | 52 |
| UNKNOWN | 9 |
| TOTAL | 916 |

severe and can sometimes be relieved in this way.

In home deliveries the doctor and midwife had to make use of the bed available, and I have watched midwives attempting to control the delivery of the head and avoid a tear with the patient on her back, the head almost buried out of sight, and compared this with the advantages of the left lateral position. The doctor can sit on the edge of the bed beside the patient with the left arm between the thighs and the left elbow on the fundus, and both hands are available to control the perineum and ease out the head, often without damage. If the patient is tired, some fundal pressure can be of real help to her and this can be done by the one person delivering by a sort of bellows action on the fundus with the left arm, nor is the position a difficult one for forceps delivery. Clearly too, in the days when we would administer simple anaesthesia with chloroform with or without ether, on an open mask, this position was far safer. If vomiting did occur as the patient came round, the danger of inhalation was minimised and over all the years I never had a case of this complication, though this is still reported to occur even after intubation by an expert anaesthetist! Nowadays it does seem that the woman's wishes are scarcely consulted, or her true comfort; the intravenous drip, the monitoring leads, the forceps deliveries all dictate the lithotomy position, regardless of back pain, sacroiliac strain or early arthritic changes in the spine. One genuine complaint today is the woman's inability to move about freely in the first stage of labour. The physiotherapists used to teach a number of positions of greatest relief in both the first and the second stages; all these old-fashioned aids to the mother seem to have been pushed aside by drip induction, monitoring and insisting on the lithotomy position. It is scarcely surprising that labour needs to be got over as quickly as possible; that analgesic drugs are more freely used; and that epidural injection is chosen more often.

**ANAESTHETICS**

I had no personal experience of spinal anaesthetics and only a few cases of pudendal block. No anaesthetics were used in 315 cases and of the rest chloroform was a great standby in the early years with or without ether, and always administered on an open mask in the left lateral position. Sometimes we were able to obtain chloroform capsules which were sufficient to ease some women through the more painful parts of labour. The various machines for administering nitrous oxide and air or oxygen were used as the years went by, aided by the C.M. attachment, which delivered the first 3 or 4 breaths of pure nitrous oxide and proved most useful.

**PAIN AND LABOUR DATA**

Table 2 shows the total births per month with a rather surprising preference for August! The exact time of labour was known in 833 cases and there seems to be some truth in the increased occurrence of birth between the hour of midnight and 4 a.m., but also it shows a rise at 5 p.m. which was one of the most awkward hours to be held up! However, I managed to be present at 808 of the 908 cases, which includes arriving within 10 minutes of birth. I did 118 forceps deliveries, of which 18 were in home confinements and the rest in general practitioner hospitals or nursing homes. Consultant advice was sought in 175 cases; there were 16 Caesarian sections and 30 breech deliveries. Induction was performed in 252 cases, 'medical' in 159 apparently successful cases, but a failure in 38 cases; surgical induction in 93 cases including those in which medical induction failed. Stitches were required in 150 cases, leaving 734 cases getting through undamaged. Haemorrhage was not a great problem. There were 92 cases of threatened miscarriage in early pregnancy which all went on successfully to term; 12 cases of placenta praevia; only one case of severe post partum haemorrhage but slight post partum haemorrhage in 73 cases; Ergot was given rountinely in 716 cases, usually, in early years, at the completion of the third stage, but, later in the series, with the anterior shoulder. Of pre-eclamptic toxaemia there were 22 cases, 16 of which were treated by induction, 5 went into labour spontaneously and 1 by Caesarian section. In all 22 cases they produced normal living babies, but there were 2 maternal deaths already referred to.

My use of drugs for pain in labour shows a strong preference for pethidine or pethilorfan (332 cases), but always in small doses of 50 to 100 mgs. Mild sedation with potassium bromide and chloral or Doriden (glutethimide) was used in 191 cases and Table 3 shows the effect of sedation on the second stage of labour. No drugs were used in 349 cases.

### BABY DATA

Finally with regard to details of the resulting babies. Of the 920, 484 were females and 406 males, 30 were unrecorded. The birth rates ranged from 2 lbs 6 ozs (1.08 kg) to 11 lbs (4.99 kg). 767 were between 5 and 10 lbs the highest number being about 8 lbs (3.63 kg). 24 were under 5 lbs (2.27 kg) and 12 were over 10 lbs (4.54 kg). There were 58 cases of 'blue asphyxia' and 34 cases of 'white asphyxia', 10 cases of stillbirth due to: post maturity 2, congenital malformations 3, prolapsed cord 2, obstructed breech delivery 1 and no cause in 2. There were 4 cases of death of the foetus earlier in pregnancy; two ceased moving, one at 7½ months and one at 5 months. One was born at full term macerated and there was one due to concealed ante-partum haemorrhage. There was success with breast feeding, 682 cases being successfully established and only 162 recorded as unsuccessful or positive refusals.

### SOME REFLECTIONS

### PAIN

It is difficult for the male doctor to talk about the pains of childbirth without being accused of callousness. However, pain is a valuable sensation and can be used under certain circumstances very

Table 3 **EFFECT OF SEDATION ON THE SECOND STAGE OF LABOUR**

| SEDATIVE OR ANALGESIC USED | LENGTH OF SECOND STAGE (HOURS) | | | | | |
|---|---|---|---|---|---|---|
| | ⩽ 1 | 2 | 3 | 4 | 5 | ⩾ 6 |
| 1. NO SEDATION | 232 | 63 | 17 | 7 | 3 | 0 |
| 2. DORIDEN (GLUTETHIMIDE) | 15 | 3 | 0 | 0 | 0 | 0 |
| 3. BARBITURATES | 5 | 5 | 3 | 0 | 1 | 1 |
| 4. POTBRUM CHLORAL | 25 | 9 | 4 | 1 | 0 | 0 |
| 5. PETHIDINE | 79 | 21 | 12 | 0 | 1 | 0 |
| 6. PETHILORFAN | 42 | 12 | 3 | 1 | 0 | 0 |
| 7. HEROIN | 2 | 2 | 0 | 0 | 0 | 0 |
| 8. MORPHINE-OMNOPON | 5 | 0 | 0 | 0 | 0 | 0 |
| 9. POT BROM + CHLORAL + NEPENTHE | 12 | 3 | 2 | 0 | 1 | 0 |
| 10. THREE FIFTEENS + PETHIDINE | 12 | 10 | 2 | 0 | 0 | 0 |
| 11. POT BROM CHLORAL + PETHIDINE | 16 | 8 | 3 | 4 | 0 | 0 |
| 12. POT BROM CHLORAL + PETHIDINE + HEROIN | 0 | 0 | 0 | 1 | 0 | 0 |
| 13. BARBITURATE + THREE FIFTEENS | 1 | 1 | 0 | 2 | 0 | 0 |
| 14. BARBITURATE, POT BROM CHLORAL + PETHIDINE | 0 | 0 | 2 | 0 | 0 | 0 |
| 15. BARBITURATE + PETHIDINE | 3 | 0 | 0 | 0 | 0 | 0 |
| 16. POT BROM CHLORAL + BARBITURATE | 4 | 0 | 1 | 2 | 0 | 0 |
| 17. DORIDEN + PETHIDINE | 5 | 0 | 4 | 0 | 0 | 0 |
| 18. DORIDEN + BARBITURATE | 2 | 0 | 0 | 0 | 0 | 0 |
| 19. DORIDEN + BARBITURATE + PETHILORFAN | 4 | 3 | 1 | 0 | 1 | 0 |
| 20. DORIDEN + PETHILORFAN | 21 | 5 | 2 | 0 | 0 | 0 |

effectively. But pain that is unneccessary or futile is truly terrible and to be fought at all costs.

The contractions of the uterus, though clearly very painful, have purpose and can produce results which give great joy. So long as the woman knows that she is making progress and all is well and that the pains are purposeful, then what is needed is sufficient help to control and contain the pains, and help the woman to 'ride the waves', as is taught by those who believe that relaxation helps.

The GP needs to 'live through' the labour with his patient, and be ready to leap in with help if progress is held up or there is anything wrong. I have come across very few women who want to be completely numb during the birth!

**SAFETY**

It is plain looking back over the years that fashions in management of labour change and recur. There are swings of opinion which make one somewhat sceptical of the modern developments in the search for safety and in medicine nothing is totally safe.

Perhaps we pay too high a price for safety in delivery today; added to some loss of the personal relationship of confidence and the waiving of the character of the patient and her own wishes, is a use of scientific apparatus which may be unnecessary in many cases. The prospective mother is a whole person and her attitudes and happiness matter greatly; she may sincerely want to deliver herself and not have it done for her. A normal labour with a successful delivery leaves the mother relaxed and satisfied; she quickly brightens up and is able to enjoy the new baby at once. The bond of love between them may be strongly influenced by her experience in labour, and by close contact as soon as the child is born.

The average time spent in labour in my series was 13.58 hours (1st stage 11.97 hrs., 2nd stage 1.4 hours, 3rd stage 0.21 hours). The young midwife today is taught to be really worried if the whole delivery is not over in 8 hours or less and this may be a high-speed forced delivery. Our high-speed trains have reduced the journey from Bristol to Paddington by about ½ hour; but if the traveller was told that this involved strapping her to her seat unable to move to buffet or toilet, with the blinds down shutting out any views, and with artificial light, she might well settle for a slower journey with more freedom.

Before *every* woman in labour is immobilised with induction drips, spinal injections, monitoring equipment, and drugs, ending in a forceps delivery with heroic episiotomy which may have unnecessary and serious after-effects; before she is robbed of what can be a rewarding experience; let it be said that there is still another comparatively safe way of having a baby with full consciousness, pain well controlled, and risks of any kind assessed by careful ante-natal observation, but as far as possible a 'natural birth', albeit with all modern aids available in cases of real risk!

**ACKNOWLEDGEMENTS**

My sincere gratitude to Professor J. R. Ashford, M.A., Ph.D. and his most capable staff in the Department of Mathematical Statistics and Operational Research at Exeter University for help and encouragement and the statistical side of this paper.

**REFERENCE**

The Management of Labour — Proceedings of Third Study Group of the Royal College of Obstetricians and Gynaecologists, 1975.

*Christopher Morgans is a retired General Practitioner from Bristol*

## Figures and Tables

**Figure 1. f1:**